# Need for multi-scale systems to identify spindle orientation regulators relevant to tissue disorganization in solid cancers

**DOI:** 10.3389/fphys.2014.00278

**Published:** 2014-07-25

**Authors:** Hui Men Selina Chin, Karandeep Nandra, Joanna Clark, Viji M. Draviam

**Affiliations:** Department of Genetics, Cancer Cell Biology, University of CambridgeCambridge, UK

**Keywords:** cell division, cancer inititation, cancer progression, spindle orientation, Hippo pathway, microtubules, Wnt pathway, PTEN regulation

## Introduction

**Figure 1:**
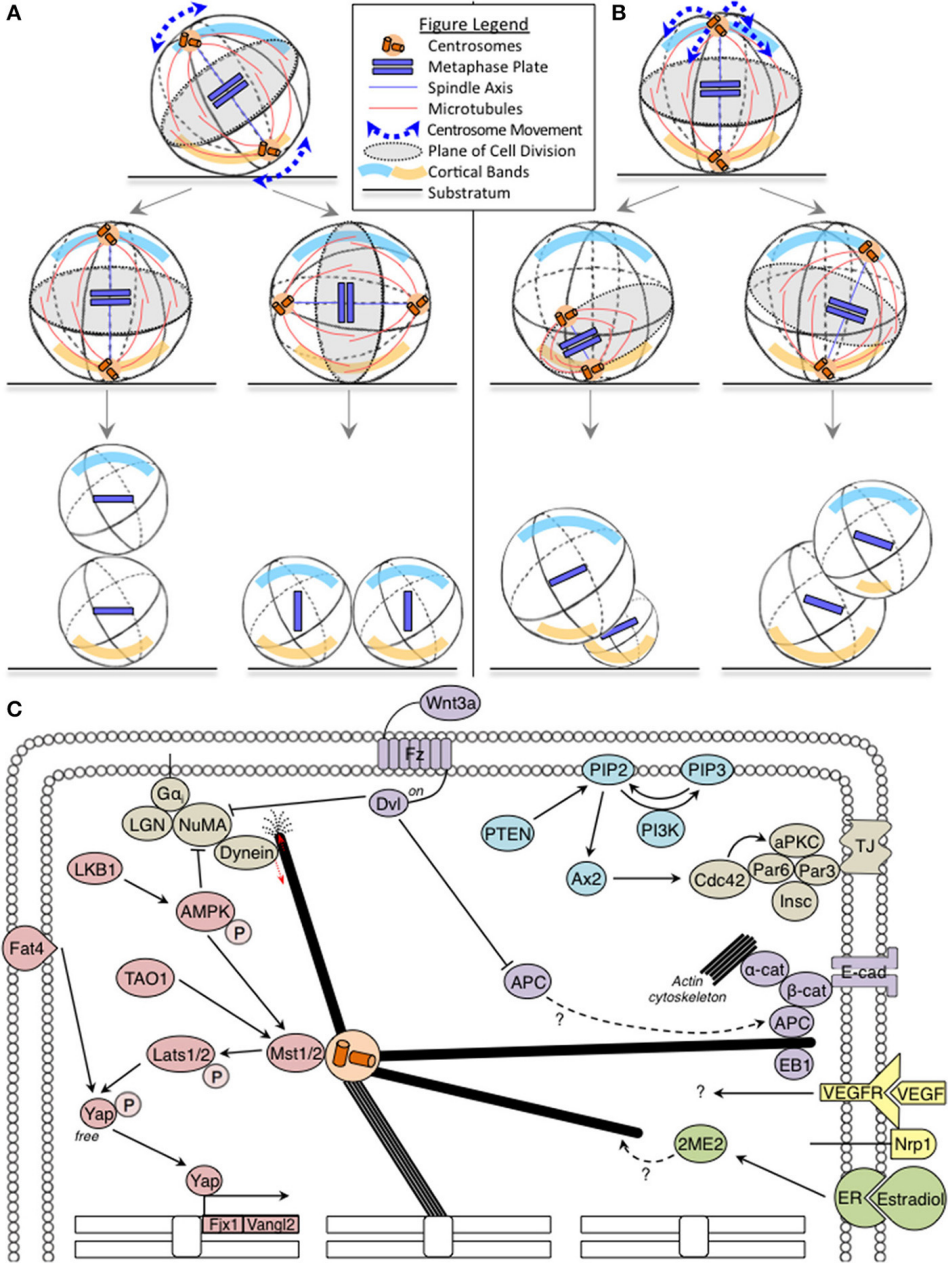
**(A,B)** Fates of incorrect spindle orientation and positioning: Cartoons show mitotic spindle movements relative to the substratum leading to spindle mis-orientation **(A)** and mis-positioning **(B)** with cortical bands highlighting polarity differences. In **(A)**, misorientation alters the relative positions and contents of daughter cells, without affecting progenitor cell sizes. In **(B)**, mispositioning affects daughter cell size, relative positions and their contents. Legend describing cell substratum, spindle microtubules, metaphase plate, and spindle movements included. **(C)** Oncogenic pathways implicated in spindle orientation: The Hippo, PTEN-PI3K, and Wnt tumor suppressor pathway components are marked in pink, blue, and purple, respectively. The oncogenic estrogen receptor (ER) pathway is marked in green. Together, these pathways regulate astral microtubule (marked in bold) function. Red arrows indicate force generation events. The Hippo pathway also influences transcriptional regulation of several genes involved in orientation (marked on chromosomes).

During cell division, the mitotic spindle captures chromosomes and segregates them into two equal sets. The orientation and position of the mitotic spindle is important because the spindle equator becomes the plane of cell division. For instance, in a columnar cell with apical and basal polarity, if the spindle pole-to-pole axis orients along the cell's long axis, the cell will divide along its short-axis; however, if the spindle axis orients along the cell's short axis, the cell will divide along its long-axis (Figure 1A). Similarly when the spindle is off-centered (mis-positioned), it results in asymmetric cell sizes in the two daughter cells, which is often used to control tissue organization (Figure 1B). Thus, errors in the orientation and positioning of the mitotic spindle can cause incorrect plane of cell division leading to incorrect cell size, content and neighborhood of daughter cells (Figures 1A,B).

A human body experiences over a trillion divisions and through age errors in cell division can accumulate; errors in spindle orientation can contribute to tissue disorganization, a hallmark of several age-related conditions and also, carcinogenesis. However, mutations in classical cortical force generators that rotate the spindle to the correct orientation have not been shown to promote carcinogenesis. In contrast, several proteins known to play a role in cancer initiation and progression are being newly identified as regulators of spindle positioning and orientation. In this opinion article, we briefly discuss the surprising lack of direct evidence for classical spindle rotation regulators in oncogenesis and present examples of oncogenic pathway components that influence spindle orientation. We conclude with the need for new strategies to uncover the contribution of spindle orientation defects to tissue disorganization commonly found in cancers and also ageing disorders.

## A weak case for cortical force generators in cancer initiation

For a detailed review on the mechanisms of spindle positioning and orientation, we recommend a recent review from Kulukian and Fuchs (2013). Astral microtubules of the spindle (Figure 1C) are pulled at and this rotates the entire spindle to a pre-defined position. Forces to pull the astral microtubules can arise from the cortex or within the cytoplasm, although classical evolutionarily conserved players have been reported at the cell cortex (reviewed McNally, 2013). Cortical pulling forces are essential for mitotic spindle positioning and orientation in human cells (see next para). Although it is very likely that compromising cortical pulling forces would lead to tissue disorganization and carcinogenesis, cortical force generator mutations are not prevalent in tumors and their genetic loss-of-function in mice do not present tumors (reviewed in Noatynska et al., 2012).

Dynein is the key player in cortical force generation and its localization is controlled by the cortical platform consisting of Gα_i_, LGN, and NuMA (Figure 1C) (Kiyomitsu and Cheeseman, 2012; Kotak et al., 2012; Corrigan et al., 2013). Considering that LGN is the primary platform for cortical dynein recruitment and absolutely essential in epithelial cells for biased rotation of the spindle (Corrigan et al., 2013), one would expect a more severe phenotype than the reported epidermal stratification defects in LGN depleted mouse skin (Williams et al., 2011). An explanation for this paradox can be gleaned from proliferation and cell death studies: First, LGN mutant mice lacking LGN's C-terminus are viable, but compromised for planar spindle orientation in the brain (Konno et al., 2008). This shows that the control of spindle orientation is essential for maintaining a population of neuroepithelial cells, but is dispensable for proliferative or differentiative decisions. In support of this idea, loss of Par3, a polarity protein that forms a complex with Par6/aPKC and controls spindle orientation (Hao et al., 2010), promotes breast tumorigenesis and metastasis, only in combination with oncogenic Notch or Ras (61L) expression (McCaffrey et al., 2012). Second, combining defects in cell death and spindle alignment disrupts epithelial integrity and causes tumor-like masses (Nakajima et al., 2013). Thus, spindle orientation defects and resulting cell fate defects could be resolved by other cell number control pathways (for example, cell proliferation and cell death), which indicates a cooperative role for orientation defects in tissue disorganization and cancer progression, rather than cancer initiation *per se*.

## Key oncogenic pathways implicated in spindle orientation

While mutations in cortical force generators present a weak case for orientation defects leading to carcinogenesis, emerging evidence show a role for oncogenic and tumor suppressor pathways in ensuring spindle orientation. Three key examples are:

**1. Hippo tumor suppressor pathway**

The Hippo pathway is disrupted in a variety of cancers (reviewed in Harvey et al., 2013). Fat4, a member of the Hippo pathway in vertebrates (Skouloudaki et al., 2009) orients the plane of cell division to maintain the planar cell polarity (PCP) of elongating tubules during kidney development and prevents cyst formation common to ageing kidneys (Saburi et al., 2008; Mao et al., 2011). Fat4 regulates the expression of Vangl2 and Fjx1 (Saburi et al., 2008), which are asymmetrically localized Wnt-Fz PCP components (Montcouquiol et al., 2006). Recent additions to the Hippo pathway, LKB1 tumor suppressor (Mohseni et al., 2014), AMPK (Thaiparambil et al., 2012) and TAO1 kinase (Poon et al., 2011) are also YAP regulators that act on Lats1 and MST2, and are important for mitosis and spindle orientation (Wojtala et al., 2011; Wei et al., 2012; Shrestha et al., 2014). It is currently unclear how Fat4, TAO1, LKB1 and the Hippo pathway link spindle orientation and tissue maintenance (Figure 1C), which is an important topic to be addressed.

**2. PTEN-PI3K signaling pathway**

The Phosphatase and tensin homolog deleted on chromosome 10 (PTEN) that regulates the PI3K-Akt-mTOR pathway are among the most frequently inactivated tumor suppressor genes in sporadic cancers (reviewed in Chalhoub and Baker, 2009). PI3K influences spindle orientation in non-polarized cells (Toyoshima et al., 2007). PTEN deficiency impairs glandular morphogenesis, through Ax2 and Cdc42, leading to abnormal multi-luminal phenotypes (Martin-Belmonte et al., 2007; Jagan et al., 2013). Thus, loss of PTEN-PI3K signaling can result in incorrectly oriented daughter cells, which may be of relevance to PTEN-associated tissue disorganization common to geriatric conditions and carcinogenesis.

**3. Wnt signaling pathway**

Multiple components of Wnt pathway are known to control spindle orientation. First, spatial restriction of Wnt3a is sufficient to align the spindle parallel to the axis of cell polarity and induce asymmetrical cell division leading to asymmetrical inheritance of Wnt signaling components. This provides a mechanism for extrinsic control of cell fate and differentiation (Habib et al., 2013), but its specific role in cancer is unclear. Second, APC, a Wnt pathway member, is a tumor suppressor and regulator of microtubule stability and cell polarity (Zumbrunn et al., 2001; Etienne-Manneville and Hall, 2003). APC and its interactors, EB1 (a microtubule-end binding protein) and β-catenin are all needed for stable spindle positioning (Draviam et al., 2006; Wu et al., 2010 and reviewed in Tamura and Draviam, 2012). While inactivation of both APC alleles is required for carcinogenesis (reviewed in Reya and Clevers, 2005), loss of a single allele is sufficient for spindle misorientation (Fleming et al., 2009). It is unclear if APC's role in spindle orientation and Wnt signaling converge in preventing carcinogenesis (Figure 1C). However, APC is known to bind β-catenin, which together with E-cadherin and α-catenin, are actin regulators with a role in spindle orientation (reviewed in Allan and Näthke, 2001). Third, Dvl is another component of the Wnt-PCP pathway which influences spindle orientation (Ségalen et al., 2010), and its role in linking spindle orientation with carcinogenesis is also not known.

In summary, studies of PTEN, Hippo and Wnt tumor suppressor pathways show evidence for more than one protein of any single pathway being involved in spindle orientation (Figure 1C). Whether their role in spindle orientation is important for their tumor suppressor function is not known and is an important question to address.

## Exciting future directions for elucidating how defective spindle orientation is linked to tissue disorganization in ageing disorders and cancers

Multiple lines of evidence show the co-existence of spindle orientation failure and growth dysregulation. Is this a mere coincidence? Alternatively, does this co-existence play any role in tissue disorganization seen in cancers or ageing disorders? To help address these questions, two approaches are going to be pivotal:

**1. Multi-scale imaging (Single-cell and tissue-level studies: two sides of a coin)**

Multi-scale systems that capture single-cell and tissue level information are crucial to track the emergence of tissue-level defects (growth dysregulation) from single-cell errors (spindle orientation failure). For instance, in cancer stem cells of skin papilloma, the inhibition of VEGF alters the ratio of symmetric:asymmetric cell divisions causing tumor regression (Beck et al., 2011). How VEGF and its co-receptor Nrp1 influence the plane of cell division is unclear (Figure 1C); and establishing this may very well require single-cell studies of the perivascular niche Cancer Stem Cells exposed to tumor-cell derived VEGF. In some cases, tissue-specific organotypic models (such as the 3D cyst model; Durgan et al., 2011) amenable to single-cell tracking may be sufficient. For example, chronic estrogen application is linked to hyperplasia and cancer: estrogen increases symmetric cell division (Gunin et al., 2001), and an estrogen metabolite, 2-methoxy estradiol (2ME), alters microtubule dynamics and disrupts spindle orientation (Corrigan et al., 2013). Determining how sub-cellular microtubule perturbation ultimately manifests into changes in symmetric vs. asymmetric cell division rates in tissues could be addressed using organotypic models that can recapitulate estradiol-dependent morphogenesis.

**2. A quantitative way to define intermediary dynamic steps of spindle orientation**

In cell cultures that have lost polarity and resemble those that have gone through Epithelial-Mesenchymal transition, even a small directional bias in moving the spindle toward the final destination is sufficient to achieve the correct orientation of the spindle (Corrigan et al., 2013)—what is the molecular basis of this robustness? Is this dependent on the microtubule -wall or -end interaction at the cell-cortex, similar to microtubule interaction geometries at chromosomes (Shrestha and Draviam, 2013)? Is this dependent exclusively on cortical pulling forces that act on microtubules or also on pushing forces of microtubules against the actin mesh-work, or forces generated by intracellular transport (reviewed in McNally, 2013). Addressing these in human cells will require us to consider the temporal evolution of various spindle movements and not simply the binary end-outcome of spindle orientation “failure” vs. “success.” Examples of similar approach have been already fruitful in *C.elegans* (Pecreaux et al., 2006; Kimura and Onami, 2010). Finally, understanding the evolution of spindle movements is important because even a simple delay in spindle movements can increase the probability of spindle orientation defects, as human cells have not been reported to have a spindle orientation checkpoint so far.

## Conclusions

Knowing the intermediary steps of the spindle orientation process can help reveal how growth regulatory pathways like the Hippo or mTOR pathway that receive various signals from developmental and stress cues, jointly regulate spindle movements. This along with multi-scale systems will be important for determining molecular lesions in spindle orientation and positioning which are frequently associated with tissue disorganization observed in ageing disorders and solid cancers.

### Conflict of interest statement

The authors declare that the research was conducted in the absence of any commercial or financial relationships that could be construed as a potential conflict of interest.
